# Brain activation during associative short-term memory maintenance is not predictive for subsequent retrieval

**DOI:** 10.3389/fnhum.2015.00479

**Published:** 2015-09-02

**Authors:** Heiko C. Bergmann, Sander M. Daselaar, Sarah F. Beul, Mark Rijpkema, Guillén Fernández, Roy P. C. Kessels

**Affiliations:** ^1^Donders Institute for Brain, Cognition and Behaviour, Radboud UniversityNijmegen, Netherlands; ^2^Department of Computational Neuroscience, University Medical Center Hamburg-EppendorfHamburg, Germany; ^3^Department of Radiology and Nuclear Medicine, Radboud University Medical CenterNijmegen, Netherlands; ^4^Department of Cognitive Neuroscience, Radboud University Medical CenterNijmegen, Netherlands; ^5^Department of Medical Psychology, Radboud University Medical CenterNijmegen, Netherlands

**Keywords:** relational memory, long-term memory, memory binding, short-term memory, neuroimaging

## Abstract

Performance on working memory (WM) tasks may partially be supported by long-term memory (LTM) processing. Hence, brain activation recently being implicated in WM may actually have been driven by (incidental) LTM formation. We examined which brain regions actually support successful WM processing, rather than being confounded by LTM processes, during the maintenance and probe phase of a WM task. We administered a four-pair (faces and houses) associative delayed-match-to-sample (WM) task using event-related functional MRI (fMRI) and a subsequent associative recognition LTM task, using the same stimuli. This enabled us to analyze subsequent memory effects for both the WM and the LTM test by contrasting correctly recognized pairs with incorrect pairs for either task. Critically, with respect to the subsequent WM effect, we computed this analysis exclusively for trials that were forgotten in the subsequent LTM recognition task. Hence, brain activity associated with successful WM processing was less likely to be confounded by incidental LTM formation. The subsequent LTM effect, in contrast, was analyzed exclusively for pairs that previously had been correctly recognized in the WM task, disclosing brain regions involved in successful LTM formation after successful WM processing. Results for the subsequent WM effect showed no significantly activated brain areas for WM maintenance, possibly due to an insensitivity of fMRI to mechanisms underlying active WM maintenance. In contrast, a correct decision at WM probe was linked to activation in the “retrieval success network” (anterior and posterior midline brain structures). The subsequent LTM analyses revealed greater activation in left dorsolateral prefrontal cortex and posterior parietal cortex in the early phase of the maintenance stage. No supra-threshold activation was found during the WM probe. Together, we obtained clearer insights in which brain regions support successful WM and LTM without the potential confound of the respective memory system.

## Introduction

Recent years have seen renewed debate and controversy over the underlying neural substrates of working memory (WM) and its (in)dependence from long-term memory (LTM). Whereas the “classical” view used to regard these two memory systems as functionally and neurally distinct (Baddeley and Warrington, 1970; Craik and Watkins, 1973; Baddeley and Hitch, 1974; Craik, 2002; Henke, 2010), more recent views stress that they are intimately linked, also with respect to their underlying neural substrate (Shallice and Warrington, 1970; Ruchkin et al., 2003; Ranganath and Blumenfeld, 2005; Mayes et al., 2007; Jonides et al., 2008; Nadel and Hardt, 2011; Olsen et al., 2012). More specifically, according to these latter views, recruitment of specific brain regions may not so much be a function of the delay between study and test, but may crucially depend on the underlying cognitive operations that need to be performed in order to execute the task at hand successfully (Jonides et al., 2008). For example, the active manipulation and updating of information may rely on dorsolateral prefrontal activation (e.g., Fuster, 1973; Kessels et al., 2000), whereas load-related activation and retrieval may rely on the posterior parietal cortex (Berryhill, 2012; Postle, 2015).

In addition to these brain regions that are typically studied in the WM literature, recent studies also suggest that the hippocampus may also be important in some aspects of WM processing. Due to its anatomical characteristics and extensive reciprocal connectivity with polymodal neocortical association areas (Suzuki and Amaral, 1994), the hippocampus plays a vital role in relational memory in general. This involvement may be unrelated to the delay length between presentation and test (Konkel and Cohen, 2009). This view appears to be both supported by recent lesion studies (Holdstock et al., 1995; Giovanello et al., 2003; Crane and Milner, 2005; Hannula et al., 2006; Nichols et al., 2006; Olson et al., 2006a,b; Hartley et al., 2007; Piekema et al., 2007; Rose et al., 2012; van Geldorp et al., 2014, 2015), intracranial EEG and MEG (Axmacher et al., 2007, 2010a,b; Cashdollar et al., 2009) and by fMRI studies (Kirwan and Stark, 2004; Ranganath et al., 2005; Nichols et al., 2006; Piekema et al., 2006, 2009, 2010; Axmacher et al., 2008, 2009; Hannula and Ranganath, 2008; Olsen et al., 2009; Oztekin et al., 2009; Schon et al., 2009; Luck et al., 2010; Libby et al., 2014) which all demonstrated hippocampal involvement in either “typical” relational WM tasks or tasks most likely involving relational binding processes (often using delayed-match-to-sample tasks).

However, one critical question concerns whether the task performance in “typical” WM tasks actually relies exclusively on WM processes rather than being also supported by LTM or WM–LTM interactions (Jeneson and Squire, 2012). In other words, depending on the kind of task, the type of stimuli, the cognitive operations required to complete the task as well as the cognitive load, performance on a WM task is, at least partially, (co-)dependent upon LTM processes rather than being a “pure” measure of WM. This, in turn, may explain why hippocampal activation is demonstrated in some fMRI studies during WM tasks and why patients with hippocampal lesions are impaired in WM tasks (Jeneson and Squire, 2012). Even though one may argue that all tasks are multiply determined (Tulving, 1991), one undoubtedly needs to control as much as possible for potential confounding factors, such as incidental LTM effects when studying WM.

Recently, Bergmann et al. (2012) minimized this potential confound by administering both an associative (pairs of faces and houses) WM task using an event-related fMRI design and a subsequent recognition memory (LTM) task, probing the same associations as during the WM task. This allowed us to isolate a “subsequent WM effect” by contrasting trials in which the pairs were correctly recognized with trials in which participants failed to correctly recognize the pairing in the WM task. Critically, we analyzed this effect exclusively for trials for which there was no evidence of successful LTM, as tested in the subsequent LTM task. This way, we obtained a clearer measure of brain activity related to successful WM processing and demonstrated that successful WM task performance was associated with increased activation in higher-order visuo-perceptual areas (i.e., parahippocampal region and fusiform gyrus) during the encoding phase. In contrast, successful LTM formation was associated with increased encoding-related activation in the hippocampus. Thus, hippocampal activation was observed during the execution of a WM task, but appeared to be more related to LTM formation rather than successful WM processing. This challenges the proposal of a critical role of the hippocampus in WM independent from long-term encoding. We therefore concluded that the distinction between what generally is referred to as WM and LTM should better be based on the underlying cognitive operations to be performed in the task (i.e., active maintenance and updating), rather than the delay between study and test.

While, Bergmann et al. (2012) isolated encoding-related activity associated with WM and LTM success, from a cognitive perspective on WM the maintenance phase is considered at least as crucial as the encoding phase, because it relies on the preservation of information in the absence of sensory stimuli—a critical aspect in almost all theoretical frameworks of WM (Baddeley and Hitch, 1974; Cowan, 1999; Miyake and Shah, 1999). Considering the relevance of the maintenance phase for WM processing and previous suggestions that the medial temporal lobe (MTL) may be activated during WM maintenance and predict LTM success (Leszczynski, 2011), we therefore modified our original design in order to analyze WM and LTM processing during the maintenance as well as the probe phase of the WM task. In the present study, we slightly modified the previously used four-pair (faces and houses) associative delayed-match-to-sample task (Bergmann et al., 2012) and administered it in the MRI scanner. This was followed by an unexpected associative recognition memory task outside the scanner. Cerebral activation during the WM maintenance and probe phases was analyzed in relation to WM and LTM performance. In addition, based on earlier reports concerning a functional heterogeneity of the WM maintenance phase we divided this phase into two separate stages: an initial stage and a late stage. That is, initially a perceptual representation is being converted into an internal code. In turn, processing during the subsequent, later phase is mainly passive maintenance of this code in the absence of external stimuli (Naveh-Benjamin and Jonides, 1984). It is the initial phase which may be critically involved in LTM formation, as it is the most effortful (Khader et al., 2007, 2010; Bergmann et al., 2013). The nature of the late stage, in contrast, may be more automatic and it is questionable whether event-related fMRI is sensitive to the underlying processes, whose temporal signature cannot be regarded as a phasic, time-locked response, but rather a tonic change in activation levels (Singer and Gray, 1995; Engel et al., 2001; Zucker and Regehr, 2002; Jensen and Lisman, 2005; Jensen, 2006; Fell and Axmacher, 2011). In an fMRI study, Ranganath et al. (2005) indeed showed that the early stage of WM processing contributed to LTM success and that this was related to prefrontal-hippocampal activation, in contrast to the late stage during which activation was identified in regions important for visual WM maintenance.

## Materials and Methods

### Participants

This study was approved by the local ethics committee (CMO region Arnhem-Nijmegen, Netherlands; #CMO2014/288). All participants provided written informed consent in accordance with the declaration of Helsinki. Thirty healthy undergraduate students (12 men; mean age = 21.83 years, ranging from 18 to 27 years, all right-handed) participated in the study. Two people (both women) were excluded from further analyses due to technical failure. Another two women were excluded because they did not have sufficient incorrect responses on the WM task (i.e., less than 10), so that this response category could not be modeled reliably. The remaining 26 participants (mean age = 21.9 years) all had normal or corrected-to-normal vision. None of the participants reported any current major medical problems or had a history of psychiatric or neurological disorders.

### Behavioral Task

**Figure 1:**
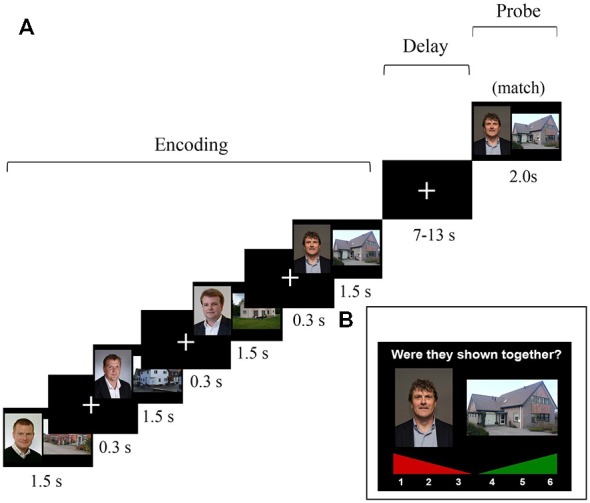
**(A)** Schematic overview of one trial of the 4-pair delayed-match-to-sample task that was administered in the MRI scanner. The probe consisted of either a match or an intra-trial re-arranged pair. Note: In the actual experiment, the slides did not cover the whole screen. The graphic stimuli where centered and depicted within a range of approximately 30° of visual angle. **(B)** An example of a trial of the self-paced subsequent recognition memory (LTM) task that was administered outside the scanner.

The task and stimuli described here were similar to those reported in Bergmann et al. (2012), with some slight modifications in the design in order to model the maintenance and probe activation. Participants performed a four-pair delayed-match-to-sample WM task in an MRI scanner (Figure 1). The stimuli presented during the study phase were colored face-house pairs that were consecutively shown; the house was always shown at the right side and the face at the left side. Presentation duration for every pair was 1.5 s, separated by a 0.3 s inter-stimulus interval in which a fixation cross was shown. This encoding period was followed by a variable 7–13 s maintenance interval, varied in steps of 2 s. Introducing a jittered delay for the maintenance period enabled us to model the neural activation during this delay (Piekema et al., 2006; Parra et al., 2014). Subsequently, one face-house pair was probed, which could be either an identical pair (“match”) or an pair that was re-arranged using a face and house that was shown in this trial, but not in that combination (“non-match”). In case of a non-match, the face was paired with a house that either preceded or followed the face. This was done to prevent ceiling performance on the WM task and to decrease the likelihood that participants based their responses upon the temporal context of the stimuli. During the probe, subjects had to indicate using a button box whether or not the presented pair was one of the four pairs that were shown during the study phase. Two-hundred trials were administered, 135 (67.5%) of which were matches. Prior to the actual experiment (outside the scanner), written instructions were given and three practice trials were performed to get acquainted with the task demands. Participants were instructed to actively maintain the stimulus pairs during the WM task, but were not informed that they would be tested again outside the scanner.

Approximately 10–15 min after having completed the WM task in the scanner, an unexpected subsequent recognition-memory test followed to test LTM for the pairs that were previously presented during the WM task in the scanner. In this recognition LTM test, face-house pairs were presented again, and participants had to indicate whether they had seen this face-house combination or not using a confidence rating scale ranging from 1 (“definitely not seen during scanning phase”) to 6 (“definitely seen during scanning phase”). Here, 200 probe trials were presented, 135 (67.5%) of which were matches. The matching/“old” probe pairs were identical to those that had been probed previously in the WM task. For the non-match probe pairs, faces and houses were used that had been individually used as probes in the WM task, but not in that combination. This was done to make sure that all faces and houses that were shown in the LTM task had also been shown in the WM task. As a result, all stimuli in the LTM task were presented once during the encoding phase and once in the probe phase of the WM task (see Figure 1 for an overview of the WM and LTM tasks).

### Image Acquisition and Data Preprocessing

A 1.5T Avanto MRI scanner system (Siemens Medical Systems, Erlangen, Germany) with a 32-channel radiofrequency head coil was used. A T1-weighted 3D MPRAGE sequence (TR = 2250 ms, TE =2.95 ms, flip angle = 15°, 176 sagittal slices, voxel size = 1 × 1 × 1 mm^3^, acquisition matrix = 256 × 256, FOV = 256 mm) was used to acquire high-resolution anatomical images. A T2*-weighted EPI sequence (TR = 2280 ms, TE = 40 ms, image matrix = 64 × 64, flip angle = 90°, slice thickness = 3.0 mm, distance factor = 10%, 32 axial slices, voxel size 3.3 × 3.3 × 3.0 mm^3^, FOV = 212 mm) was used to collect whole-brain functional images. In order for the magnetization to approach a dynamic equilibrium the first five volumes of the EPI series were not included in the analysis. In subsequent data processing, functional EPI-BOLD images were realigned using a six-parameter, rigid-body transformation algorithm. Next, mutual information optimization was used to co-register the mean of the functional images to the structural MR image. Subsequently, functional images were spatially normalized, re-sampled to create 3 mm isotropic voxels and transformed into a common stereotactic space (as defined by the SPM5 MNI T1 template). Finally, an 8 mm FWHM Gaussian filter was used to spatially smooth the images. To remove low-frequency drifts from the data the highpass filter was set to the SPM default of 128 s.

### fMRI Data Analysis

Statistical parametric mapping using SPM5 software was performed to analyze the fMRI results (Wellcome Department of Cognitive Neurology, London, UK) using the general linear model (GLM). Goal of the present study was to examine which brain-region activation was predictive for a successful performance on the WM and LTM task during the initial stage and later stage of the WM maintenance phase, as well as the probe phase of the WM task. Consequently, we focused our analysis on match pairs only (pairs probed in both the WM task and the LTM task). The memory performance was used to divide trials into four response categories, since participants could either have identified study pairs correctly (referred to as “hits”) or incorrectly have classified them as being a new, re-arranged pair (“misses”) on both the WM and LTM task. As a result, the following four categories were possible, as described previously in Bergmann et al. (2012): (1) WM hit/LTM hit (referred to as WM+/LTM+); (2) WM hit/LTM miss (WM+/LTM−); (3) WM miss/LTM hit (WM−/LTM+); and (4) WM miss/LTM miss (WM−/LTM−). It must be noted that WM−/LTM+ responses occurred only in 3.2% of the trials, which resulted in insufficient power. This combination was therefore used as a regressor of no-interest. The other three categories were used as separate regressors of interest as a function of the WM phase. Participants with fewer than ten trials in one or more of the three categories were excluded from further analyses. This is similar to our previous study, which resulted in sufficient statistical power to obtain statistically reliable results (Bergmann et al., 2012).

**Figure 2:**
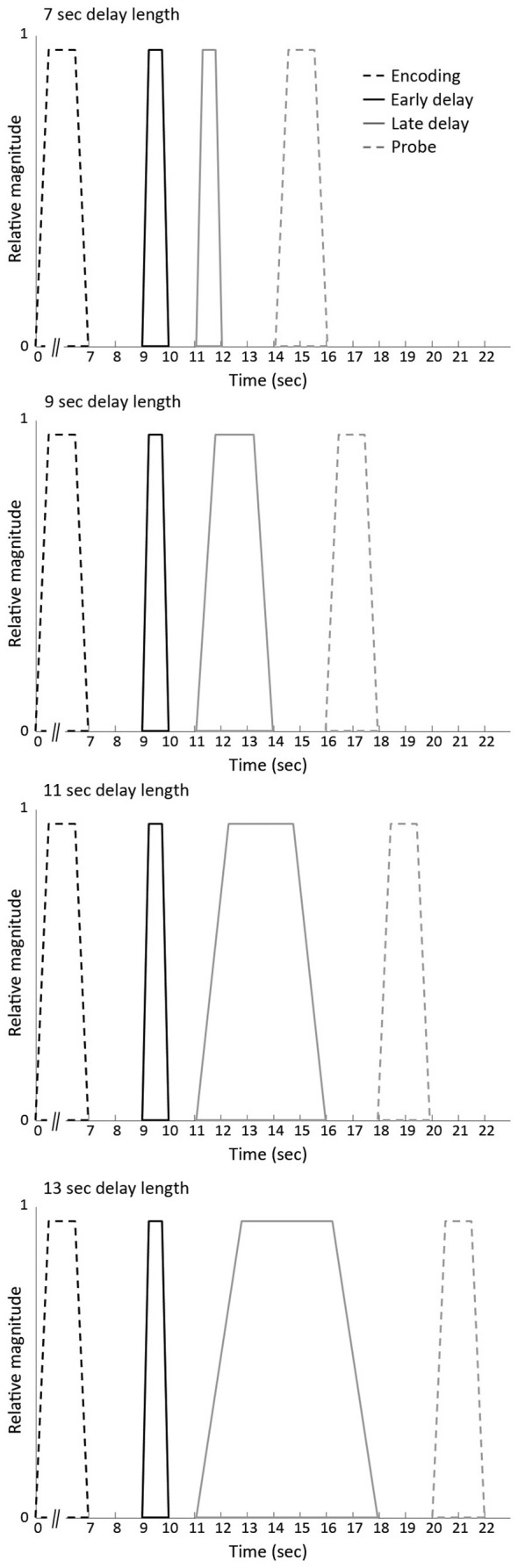
**Vectors of expected neural activity corresponding to early delay, late delay and probe phase**. Covariates modeling BOLD response on each working memory (WM) trial were constructed by convolving the different stages (i.e., early delay, late delay or probe phase) with its respective duration and convolved with the canonical hemodynamic response function.

The identical vector definition (i.e., onset, duration and expected neural activity that was associated with each component) as implemented by Ranganath et al. (2005) was used (see Figure 2): the construction of the covariates for early and late stage of WM maintenance was based on the expectation that processing associated with the initial stage would take place in the first few seconds of the maintenance phase. Processing related to the late stage of WM maintenance, in contrast, was suggested to persist throughout the remainder of the WM maintenance phase. To minimize the likelihood that activity overlapped with other WM stages, onset and offset of the early and the late stage of the delay phase were spaced apart from each other as well as from the probe phase.

The events of the three response categories were modeled by time-locking the onset of either the early delay, late delay or probe phase with its respective duration (i.e., 1 s for the early delay, variable duration for the late delay and 2 s for the probe) and convolved with the canonical hemodynamic response function. The remainder of the encoding events (i.e., unprobed pairs or pairs to which participants did not respond) were considered regressors of no interest in the model.

### Second-Level Analysis

Second-level factorial analysis was performed on the created individual contrast images, which consisted of two factors: (1) Phase, consisting of three levels (early delay, late delay and probe phase); and (2) Response Category, comprising the three levels of interest (WM−/LTM−, WM+/LTM−, WM+/LTM+). Subjects were considered random variables. An uncorrected threshold of *p* = 0.001 was first applied to the results of the random effects analyses. Note that we did not examine the other tail of the contrast (i.e., Misses > Hits) as the focus of the present paper was on subsequent memory effects, that is, the Hits > Misses contrasts.

Next, we used cluster-size statistics as test statistic. *p*FWE < 0.05 (FWE corrected for multiple non-independent comparisons; Worsley et al., 1996) was considered significant for clusters for the whole-brain analyses, and their local maximum’s MNI coordinates were recorded. Furthermore, we created an anatomical region of interest (ROI) which bilaterally included the hippocampus or the parahippocampal region, respectively, because of the assumed involvement of the MTL. Considering the fact that prefrontal as well as parietal areas are traditionally associated with WM maintenance and that these two regions have been implicated in the WM “core network” (Rottschy et al., 2012), we defined two more ROIs which either bilaterally covered the frontal lobes or bilateral inferior and superior parietal lobes (using the WFU Pick Atlas) to be applied as a small-volume correction mask (at *p*SVC < 0.05).

## Results

The results section is organized similarly as in Bergmann et al. (2012), now reporting the current behavioral and brain activation results for the early and late delay phase separately.

### Behavioral Data

#### WM Task

Mean hit rate was 72.7% (±12.48) and mean false alarm rate 41.1% (±11.14), d’ = 0.87, ± 0.44. In 3.69% (±4.01) of the trials subjects did not respond within the set windows of 2 s.

#### LTM Task

**Figure 3:**
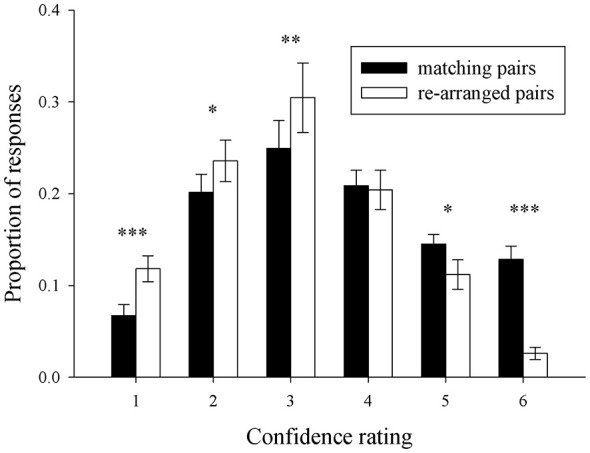
**Behavioral performance on the subsequent recognition memory (LTM) task**. Distributions of mean hit and false alarm rates: Mean (±SEM) proportions of responses are depicted on the *y*-axis and confidence ratings (“1”: definitely a re-arranged pair; “6”: definitely a matching pair) on the *x*-axis. ***p < 0.001, **p < 0.01, *p < 0.05.

Figure 3 shows the distribution of mean response proportions in the LTM task. A 2 (stimulus type: match vs. re-arranged) by six (confidence rating: 6 levels) repeated-measure MANOVA demonstrated a significant interaction between confidence rating and stimulus type, *F*_(5,93)_ = 23.72, *p* < 0.0005, ${\text{η}}_{p}^{2}$ = 0.49. “Six” and “five” ratings occurred more frequently for match pairs than for non-match trials (*post hoc* paired-sample analyses: *t*_(25)_ = 7.84, *p* < 0.0005 and *t*_(25)_ = 4.32, *p* < 0.0005, respectively). In turn, “1”, “2” and “3” ratings occurred less often for match pairs than for re-arranged pairs (*t*_(25)_ = 6.08, *p* < 0.0005; *t*_(25)_ = 3.25, *p* = 0.003; *t*_(25)_ = 3.89, *p* = 0.001, respectively). There was no differences between match- and non-match trials for “4” ratings (*t* < 1). Additional individual analyses revealed that more than 60% of the participants more often gave a “4” rating for re-arranged pairs than for old pairs. In addition, visual inspection of the “3” and “4” responses for re-arranged pairs (the white bars in Figure 3) shows that more “3” responses were given than for “4” responses for “old” pairs. These findings indicate that the participants could discriminate between presented and re-arranged face-house pairs at all confidence levels, apart from confidence rating “4”. Therefore, LTM “hits” were defined as correctly endorsing an intact pair with a confidence rating of 5 or 6. In contrast, trials in which participants failed to endorse intact pairs with a “positive” rating (i.e., 1–3) as well as the “4” responses were categorized into a separate bin. Hence, for the LTM task we contrasted trials where participants were able to build a clear and strong memory for the probed pairs with trials of incorrect or weak memory traces. Each participant had more than 10 events of each response category.

#### LTM in Relation to WM Task

In addition, we computed the conditional probabilities of LTM memory success given WM success or failure. As can be seen in Table 1; the likelihood of correctly recognizing an old pair in the LTM task is profoundly reduced when the pair was not correctly identified in the previous WM task (e.g., only 2% confidence “6” ratings for previously incorrect pairs as compared to 18% for previously correct pairs).

**Table 1:** **Behavioral performance (proportion of responses) on the LTM task as a function of the performance on the WM task**.

Response in LTM task	When WM was …	
	Incorrect	Correct
1 (“definitely a re-arranged pair”)	0.07	0.07
2	0.24	0.18
3	0.34	0.23
4	0.23	0.20
5	0.10	0.15
6 (“definitely a match”)	0.02	0.18

### Functional Imaging Data

Table 2 shows an overview of the conducted functional imaging analyses. Identical analyses were performed for all three defined stages (early delay, late delay and probe phase).

**Table 2:** **Overview of the conducted fMRI analyses**.

Phase	Subsequent WM effects …		Subsequent LTM effects …	
	Irrespective of LTM performance	Equating for LTM performance	Irrespective of WM performance	Equating for WM performance
Early delay, late delay, and probe	(WM+ > WM−)	(WM+/LTM− > WM−/LTM−)	(LTM+ > LTM−)	(WM+/LTM+ > WM+/LTM−)

#### Subsequent Memory Effects Irrespective of WM or LTM Performance

##### Early and late delay phase

*Subsequent WM effect irrespective of LTM performance.* First we contrasted WM hits (irrespective of LTM success) with WM misses (i.e., (WM+/LTM+ and WM+/LTM−) > WM−/LTM−) for the early and late stage of the WM maintenance phase separately. However, no activation clusters survived the cluster correction or the small volume corrections for hippocampus or parahippocampal region for either stage (see Table 3).

**Table 3:** **Early (1) and late (2) maintenance-related activations for the subsequent WM effect irrespective of LTM performance and (3) early and (4) late maintenance-related subsequent LTM effect irrespective of WM performance**.

Brain region	BA	Cluster size	*t*-value	*z*-value	MNI		
					*x*	*y*	*z*
*(1) WM irrespective of LTM performance (WM+ > WM−): Early delay*
—no suprathreshold clusters—							
*(2) WM irrespective of LTM performance (WM+ > WM−): Late delay*
—no suprathreshold clusters—							
*(3) LTM irrespective of WM performance (LTM+ > LTM−): Early delay*
Left dorsolateral prefrontal cortex	L 46	51	4.44^*a*^	4.34	−30	45	33
*(4) LTM irrespective of WM performance (LTM+ > LTM−): Late delay*
—no suprathreshold clusters—							

*^a^*p*_FWE_ = 0.059*.

**Figure 4:**
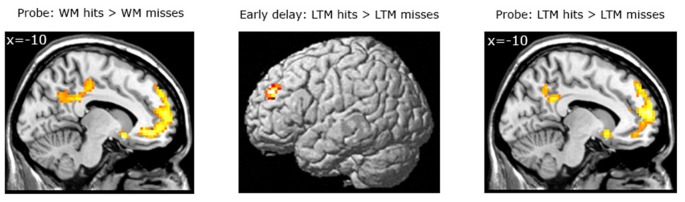
**Left panel:** Brain areas related to successful WM processing during the WM probe phase, irrespective of LTM performance (WM+ > WM−). A correct decision during the WM probe phase was associated with greater activation in a “core memory retrieval network” including the medial prefrontal cortex, posterior cingulate cortex and precuneus. **Middle and right panels**: Brain areas related to successful LTM formation during either the early WM maintenance (**middle panel**) or WM probe phase (**right panel**), irrespective of WM performance (LTM+ > LTM−). Activation in the left dorsolateral prefrontal cortex predicted LTM success during the early delay. A highly overlapping activation pattern (compared to the WM task) was found for the probe phase. Activation clusters (*p* < 0.001, uncorrected, >25 voxels) superimposed on averaged (*n* = 26) high-resolution T1-weighted images. Note: R = right.

*Subsequent LTM effect irrespective of WM performance.* When contrasting LTM hits and LTM misses irrespective of WM performance (WM+/LTM+ > (WM+/LTM− and WM−/LTM−)) for the early stage of WM maintenance, one region in the left dorsolateral prefrontal cortex showed marginally significant activation (local maximum at (−30, 45, 33); *p*FWE = 0.059; see Figure 4; Table 3). No additional clusters exhibited suprathreshold activation after small volume correction for either hippocampus or parahippocampal region. In addition, a similar analysis for the late delay stage did not reveal brain regions exhibiting suprathreshold activation.

##### Probe phase

*Subsequent WM effect irrespective of LTM performance.* Similar analyses were performed for the WM probe phase (see Figure 4; Table 4). As for the WM contrast, this revealed greater activation for WM hits vs. WM misses in the medial prefrontal cortex (local maximum at (−6, 42, −9); *p*FWE < 0.001), the posterior cingulate cortex extending into the precuneus (local maximum at (−3, −54, 33); *p*FWE < 0.001), the precentral gyrus/M1 (local maximum at (48, −18, 60); *p*FWE = 0.015) and two regions in the left middle temporal gyrus: an anterior part (local maximum at (−60, −21, −18); *p*FWE = 0.013) and a posterior part (local maximum at (−60, −57, −3); *p*FWE = 0.038). Small-volume corrections for the hippocampus or parahippocampal region did not result in additional clusters to be activated.

**Table 4:** **Probe-related activations for (1) the subsequent WM effect irrespective of LTM performance and (2) the subsequent LTM effect irrespective of WM performance**.

Brain region	BA	Cluster size	*t*-value	*z*-value	MNI		
					*x*	*y*	*z*
*(1) WM irrespective of LTM performance (WM+ > WM−)*
Medial prefrontal cortex	10/11	638	5.64^*a*^	5.45	−6	42	−9
			5.41	5.23	−3	54	−3
			5.33	5.16	−6	63	9
Posterior cingulate cortex/precuneus	23	210	4.45^*a*^	4.36	−3	−54	33
			4.24	4.15	−9	−36	30
			3.88	3.81	−9	−27	42
Right Pre-/postcentral gyrus	R 3	73	4.23^*a*^	4.14	48	−18	60
			3.71	3.65	42	−24	66
Left middle temporal gyrus	L 20	76	4.89^*a*^	4.76	−60	−21	−18
			4.37	4.28	−51	−15	−18
Left middle temporal gyrus	L 37	58	4.21^*a*^	4.13	−60	−57	−3
			3.53	3.48	−51	−75	3
			3.52	3.47	−57	−51	−9
*(2) LTM irrespective of WM performance (WM+ > WM−)*
Left hippocampus		2	3.54^*b*^	3.49	−27	−24	−15
Left parahippocampal region	L 28	3	4.00^*b*^	3.93	−21	−3	−27
Medial Prefrontal Cortex	10/11	396	5.48^*a*^	5.31	−15	60	24
			5.36	5.19	−9	60	15
			5.35	5.19	0	48	−18
Posterior cingulate cortex/precuneus	23	147	4.40^*a*^	4.31	−9	−45	30
			4.37	4.28	15	−51	36
			4.18	4.10	3	−48	39
Left middle temporal gyrus	L 21	210	4.92^*a*^	4.79	−60	−21	−15
			4.43	4.33	−60	−42	−6
			4.10	4.02	−45	−21	−9

*^a^*p*_FWE_ < 0.05; ^b^*p*_SVC_ < 0.05*.

*Subsequent LTM effect irrespective of WM performance.* A similar analysis for the LTM contrast during the WM probe (see Figure 4; Table 4) phase showed greater activation in the left hippocampus (local maximum at (−27, −24, −15); *p*SVC = 0.05) and in the left parahippocampal region (local maximum at (−21, −3, −27); *p*SVC = 0.013). Outside the MTL, an activation pattern within the anterior and posterior midline was found. That is, in the medial prefrontal cortex (local maximum at (−15, 60, 24), *p*FWE < 0.001) and posterior cingulate cortex extending into the precuneus (local maximum at (−9, −45, 30); *p*FWE < 0.001). In addition, we found greater activation in the middle temporal gyrus in the left hemisphere (local maximum at (−60, −21, −15); *p*FWE < 0.001).

#### Subsequent Memory Effects Equating for Either WM or LTM Performance

##### Early and late delay phase

*Subsequent WM effect equating for LTM performance.* To take into account possible contamination effects of LTM when estimating WM effects, we investigated which areas in the brain were recruited specifically for WM hits in comparison to WM misses for the trials in which no successful or only weak LTM formation was present, that is, WM+/LTM− > WM−/LTM−. However, neither stage of the WM maintenance phase revealed differential activation (see Table 5).

**Table 5:** **Activations for the subsequent WM effect equating for LTM performance (WM+/LTM− > WM−/LTM−) during early (1) or (2) late stage of the WM maintenance phase and the subsequent LTM effect equating for WM performance (WM+/LTM+ > WM+/LTM−) for (3) early and (4) late delay**.

Brain region	BA	Cluster size	*t*-value	*z*-value	MNI		
					*x*	*y*	*z*
*(1) WM equating for LTM performance (WM+/LTM− > WM−/LTM−): Early delay*
—no suprathreshold clusters—
*(2) WM equating for LTM performance (WM+/LTM− > WM−/LTM−): Late delay*
—no suprathreshold clusters—
*(3) LTM equating for WM performance (WM+/LTM+ > WM+/LTM−): Early delay*
Left dorsolateral prefrontal cortex	L 46	105	4.92^*a*^	4.79	−30	45	30
			3.72	3.66	−21	39	36
			3.66	3.61	−36	27	33
Left posterior parietal cortex	L 40	27	4.38^*b*^	4.28	−57	−42	51
*(4) LTM equating for WM performance (WM+/LTM+ > WM+/LTM−): Late delay*
—no suprathreshold clusters—

*^a^*p*_FWE_ < 0.05; ^b^*p*_SVC_ < 0.05*.

**Figure 5:**
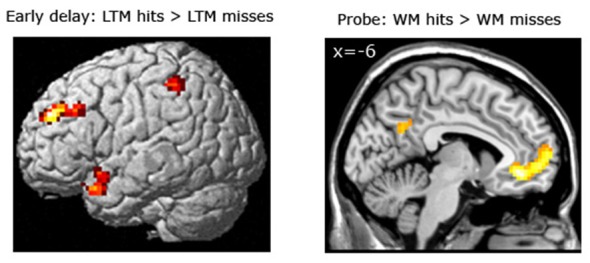
**Left panel:** Brain areas related to successful LTM formation during the early WM maintenance phase **(bottom left panel)**, equated for WM performance (WM+/LTM+ > WM+/LTM−). Successful LTM formation during the early WM delay phase was associated with greater activation in the left dorsolateral prefrontal cortex, left posterior parietal cortex/intraparietal sulcus and left temporal pole (the latter did not survive a multiple comparison correction). **Right panel**: Brain areas related to successful WM processing during the WM probe phase (top right), equated for LTM performance (WM+/LTM− > WM−/LTM−). A correct WM decision was associated with greater activation in the medial prefrontal cortex and precuneus (the latter did not survive a multiple comparison correction, though). Activation clusters (*p* < 0.001, uncorrected, >25 voxels) superimposed on averaged (*n* = 26) high-resolution T1-weighted images. Note: R = right.

*Subsequent LTM effect equating for WM performance.* For the LTM task, brain areas were identified that predicted strong LTM performance for pairs which had been correctly classified during the WM task (see Figure 5; Table 5). Here, we contrasted correctly recognized pairs in the WM task which were also correctly identified in the LTM task (with high confidence ratings) with pairs which were correctly identified during the WM task but not in the LTM task (i.e., WM+/LTM+ > WM+/LTM−). For the early maintenance phase, this analysis revealed greater activation in the left dorsolateral prefrontal cortex (local maximum at (−30, 45, 30); *p*FWE = 0.003) and left posterior parietal cortex/intraparietal sulcus (local maximum at (−54, −42, 55); *p*SVC = 0.022). As for the late stage of the WM maintenance phase, no brain regions exhibited differential activation for LTM hits vs. misses.

##### Probe phase

*Subsequent WM effect equating for LTM performance*. A correct WM decision during probe was associated with increased activation in the medial prefrontal cortex (local maximum at (−6, 39, −6); *p*FWE < 0.001). Small-volume corrections for the MTL or parietal areas did not reveal additional significant voxels (see Table 6).

**Table 6:** **Probe-related activations for (1) the subsequent WM effect equating for LTM performance (WM+/LTM− > WM−/LTM−) and (2) the subsequent LTM effect equating for WM performance (WM+/LTM+ > WM+/LTM−)**.

Brain region	BA	Cluster size	*t*-value	*z*-value	MNI		
					*x*	*y*	*z*
*(1) WM equating for LTM performance (WM+/LTM− > WM−/LTM−)*							
Medial prefrontal cortex							
	10/11	321	5.38^*a*^	5.21	−6	39	−6
			4.69	4.58	−3	54	−3
			4.57	4.47	12	60	0
*(2) LTM equating for WM performance (WM+/LTM+ > WM+/LTM−)*
—no suprathreshold clusters—

*^a^*p*_FWE_ < 0.05*.

*Subsequent LTM effect equating for WM performance.* Similar analyses for the subsequent LTM effect during the WM probe phase yielded no significant activation, given our statistical threshold, given our statistical threshold.

## Discussion

The current results on the underlying neural substrates of successful WM and LTM during the maintenance and WM probe phase extend previous findings focusing on the encoding phase (Bergmann et al., 2012). Previous studies typically studied either WM or LTM in isolation and did not consider potentially contaminating effects of incidental LTM formation processes during different WM stages (i.e., encoding, maintenance and probe). The present study examined which brain areas sustain successful WM processing when LTM fails, as well as which areas underlie the building of strong LTMs for stimuli which had already been successfully processed in WM. That is, by controlling for LTM performance, we reduced the likelihood of potential confounding effects and obtained a clearer measure of brain regions and networks supporting successful processing in an associative WM task. Results for the WM contrast “corrected” for LTM performance (i.e., contrasting WM task hits with WM task misses for pairs later forgotten in the LTM task) showed that, not unexpectedly (see “Discussion” Section below), no clusters survived the statistical threshold for the WM maintenance period. In contrast, an established “retrieval-success network” (Henson et al., 2005; Wagner et al., 2005; Buckner et al., 2008; Huijbers et al., 2010), comprising anterior and posterior midline brain regions, was activated during the probe phase. With respect to the LTM contrast, equating for WM performance (i.e., contrasting high-confidence LTM task hits with LTM task misses or low-confidence hits for pairs which were processed correctly in the previous WM task), activation in the dorsolateral prefrontal cortex and posterior parietal cortex/intraparietal sulcus in the left hemisphere during the early stage of the WM maintenance phase predicted performance on the LTM task. Finally, no clusters exhibited suprathreshold activation for the subsequent LTM effect during the late delay or the WM probe phase. The results are discussed in more detail below.

### Maintenance Phase

The idea that persistent neural activity underlies active online WM maintenance has been postulated decades ago. For example, the first electrophysiological evidence in monkeys in favor of this neural basis of WM maintenance has been reported in the early 1970s (Fuster and Alexander, 1971; Fuster, 1973) and in monkey lesion studies as early as in the 1930s (Jacobsen, 1935). However, persistent activity within isolated brain regions is most likely not the underlying mechanism of active information maintenance (Gazzaley et al., 2004). Rather, complex interactions between distributed nodes of neural networks, possibly via transient changes in synaptic efficiency (Zucker and Regehr, 2002) or neuronal populations oscillating synchronously (Singer and Gray, 1995; Engel et al., 2001; Jensen and Lisman, 2005; Jensen, 2006) are thought to subserve active WM maintenance. Hence, considering the proposed underlying mechanisms as well as the fact that Hannula and Ranganath (2008) in their subsequent WM analyses also failed to demonstrate brain regions exhibiting suprathreshold activation during maintenance, it is not surprising that we also did not succeed in finding maintenance-related suprathreshold activation for the WM accuracy contrast. In addition, it is possible that no differential maintenance-related activation was found between pairs correctly recognized and pairs not correctly recognized as the WM load for successful and unsuccessful trials was comparable and participants in both conditions did actively maintain the to-be-remembered information. However, they may have failed to maintain the correct associations of faces and houses (i.e., possibly due to incorrect forming of associations during the encoding phase) or did not correctly retrieve them during the probe phase.

One may object, though, that a number of previous studies were able to show persistent activity in different brain regions during WM maintenance (see review by Ranganath, 2006). It is important to note, however, that most of these studies either contrasted the WM maintenance period with low-level resting baseline, sensory-motor control tasks, or analyses were based on performance on a subsequent LTM task (i.e., a subsequent LTM effect), a substantial difference with the present study that investigated which brain areas supported successful execution of a WM task while holding other aspects of the other memory task constant.

The fact that it makes a difference whether one analyzes a subsequent WM or LTM effect is also demonstrated in our subsequent LTM analysis (i.e., LTM hits vs. LTM misses), exclusively for pairs previously correctly recognized in WM. During the early stage of the WM maintenance phase, high-confident LTM accuracy was associated with greater activation in brain regions traditionally proposed to play an important role during WM maintenance: the left dorsolateral prefrontal cortex (Miller et al., 1966; Fuster and Alexander, 1971; Kubota and Niki, 1971; Kojima and Goldman-Rakic, 1982; Courtney et al., 1998), left posterior parietal cortex/intraparietal sulcus (Gnadt and Andersen, 1988; Koch and Fuster, 1989; Snyder et al., 1997; D’Esposito et al., 1999; Curtis et al., 2004) as well as the specific fronto-parietal synchronous interaction (Hebb, 1949; Oliveri et al., 2001; Payne and Kounios, 2009; Fell and Axmacher, 2011; Salazar et al., 2012; see also meta-analysis by Rottschy et al. (2012); which identified a fronto-parietal network commonly activated across fMRI studies). Engagement of both brain regions has been suggested to reflect executive control and attentional processes, and sustaining the firing pattern during the maintenance phase in order to build-up an episodic representation (Ranganath, 2006). Particularly the dorsolateral prefrontal cortex may be important for the modulation of activity in posterior cortical areas and in engaging executive control mechanisms that allow for manipulation of, comparisons across, and the selection from representations being maintained in WM (Davachi et al., 2001; Wagner et al., 2001; Hopf et al., 2006), particularly in relational memory tasks (Mitchell et al., 2000; Prabhakaran et al., 2000; Piekema et al., 2006; Murray and Ranganath, 2007; Hannula and Ranganath, 2008). Our findings further suggest that engagement of the dorsolateral prefrontal cortex may only play a temporary role during the maintenance phase, i.e., during the early stage of WM maintenance, rather than persistently across the whole maintenance phase. As mentioned previously, during the initial stage of the maintenance phase an internal representation of the target is thought to be formed (Ranganath and Blumenfeld, 2005) and information active in WM needs to be organized, as in our study, in which a total of four faces, each associated with a corresponding house, had to be remembered correctly. Considering the relatively high load as well as high pace of the encoding phase, a re-organizing of the pairs may have been necessary. In their review, Blumenfeld and Ranganath (2007) proposed the dorsolateral prefrontal cortex is important for associating multiple items in WM, thereby enhancing and strengthening LTM formation for this kind of information. The notion that this may be particularly crucial for LTM formation can also be seen in our analyses of the LTM where we did not consider WM performance (i.e., the “classical” subsequent memory effect LTM+ > LTM−) and which largely failed to demonstrate fronto-parietal activation.

One also needs to consider that the delay phase was not specific for only the pair that was tested in the WM and/or LTM task. Rather, all four face house pairs seen in a given trial may have possibly contributed to delay-period activity. In future studies this issue could be addressed in more detail, for instance, by probing more pairs than just one and performing parametric analyses with the amount of correct responses as the outcome variable. However, in Bergmann et al. (2012), we did probe three pairs in each WM trial. However, we were not able to perform parametric analyses in study, because 0 or 1 correct responses were hardly observed. As a result, the number of trials per bin were unequally balanced.

### Probe

At retrieval, larger activation in brain areas previously described as being part of a generic, content-independent “retrieval-success network” was associated with a correct WM decision (Henson et al., 2005; Wagner et al., 2005; Buckner et al., 2008; Huijbers et al., 2010), including the posterior midline region consisting of the the precuneus, retrosplenial cortex and posterior cingulate; note that activation in these areas did not survive a multiple comparison correction in the present paper for the “corrected” WM contrast), the medial prefrontal cortex as well as the hippocampus. With respect to the hippocampus, hippocampal activation may be modulated by delay length, with retrieval after shorter delays may depend on the hippocampus to a lesser extent than retrieval after longer delays (Brozinsky et al., 2005; Talmi et al., 2005; Huijbers et al., 2010). Thus, it is not surprising that we did not find hippocampal involvement in our analysis. Interestingly, however, the retrieval success network has been shown to be important for episodic or LTM retrieval rather than successful WM retrieval (Henson et al., 2005; Wagner et al., 2005; Buckner et al., 2008; Huijbers et al., 2010). Hence, greater activation of these brain regions may suggest that the allotted time constraint of 2 s, within which our participants had to respond, was sufficient to allow for controlled strategic retrieval processes. This may seem at variance with the notion that information is “active” in WM, i.e., in the focus of attention (Cowan, 1999) and hence, that no strategic processes should be required to actively “retrieve” the to-be-learned information. However, the latter view may be particularly true for relatively “simple” memoranda that have to be actively maintained only (rather than transformed or manipulated), and which are tested after a delay of not more than a few seconds. As soon as the information becomes more complex (Jeneson and Squire, 2012), the cognitive load (Schon et al., 2009) or the delay length increases (Brozinsky et al., 2005; Talmi et al., 2005; Huijbers et al., 2010), and/or the information needs to be transformed or manipulated (a core feature of “working” memory in contrast to “short-term memory”), more complex cognitive operations may be required in order to make an accurate WM decision and hence, information may need to be actively retrieved.

The analysis of the subsequent LTM effect, in contrast, did not reveal any reliable effects. Only a marginally significant activation was found in left hippocampus after a small-volume correction, possibly because the WM probe phase may have served as a second encoding event for the subsequent LTM task (the identical pairs were examined later in the LTM task, and encoding of these associations has previously been reported to be hippocampus-dependent (Bergmann et al., 2012). This (relative) null-finding can be explained by the fact that participants were not aware that a subsequent LTM task would follow the WM task. As a result, there was no reason to recruit additional strategic processes for remembering the association on the long term.

### Conclusion

The present study is one of the first to disentangle brain regions supporting WM performance and brain regions involved in the building of strong LTMs during the execution of a WM task. This approach already revealed insights concerning encoding-related activity associated with successful WM or LTM (Bergmann et al., 2012). Here, we extended this finding by investigating the maintenance and probe phase of an associative delayed-match-to-sample task. Interestingly, maintenance-related engagement of a fronto-parietal network (the WM “core network”; Rottschy et al., 2012) was found to be particularly associated with successful LTM formation rather than WM. This does not necessarily imply that activation of this network does not support WM task performance. However, it may suggest that successful execution of an associative WM task may be relatively stronger supported by mechanisms to which fMRI is rather insensitive. For example, neuronal oscillations may underlie the short-term retention of information outside the focus of attention, which cannot be measured with current fMRI techniques (Postle, 2015). Alternatively, processes during encoding and the probe phase may be more critical determinants of WM accuracy. Postle (2015) argues, for instance, that delay-period activity may reflect attentional or encoding-related processes that may be ongoing even in the absence of the stimuli rather than short-term retention or maintenance. Concerning the probe phase, we found a core retrieval success network, previously proposed to be implicated in episodic/LTM retrieval, suggesting that strategic retrieval processes may also be involved in this kind of associative WM task.

With respect to the task used and the processes involved, one might argue that our paradigm does not assess WM maintenance as such, but is basically a short-delay LTM paradigm. Larocque et al. (2014) address this issue, arguing that disentangling the neural correlates of WM and LTM is challenging, as both WM and LTM are constructs that are defined in behavioral terms. Consequently, neural claims regarding the underlying mechanisms of WM and LTM can be subject to circular reasoning. They argue that while weight- or activity-based neural mechanisms provide insight into the neurophysiological workings of memory, they basically can only be used to support state-based models of short- and long-term retention (cf. Ranganath and Blumenfeld, 2005; Henke, 2010). From this perspective delay-related activation during our paradigm may basically reflect ongoing attentional processing that—while diminishing in the absence of external stimuli—can be reactivated after short delays without the need for active maintenance (Postle, 2015).

In contrast, system-based perspectives on memory (such as Baddeley and Hitch, 1974 model) argue that different stores for WM and LTM exist, each with its own neural substrate (Larocque et al., 2014). From a system-based perspective, it can be argued that the to-be-remembered information in our paradigm (i.e., four combinations of faces and houses) may exceed the limited capacity of the WM store, making it a LTM task. However, it should be noted that the item information itself (i.e., the faces and houses) did not have to be maintained, as both target and lure probes were always (re)pairings of the items shown in that trial. While maintaining the combination of faces and houses is challenging given the visual nature of the task, participants reported that they were actively and verbally associating the faces and the houses during the WM task. We argue that this indicates that participants were actively maintaining the stimuli using WM processes. If participants simply waited until the probe pair appeared without effortful processing during the delay, we would also expect the behavioral performance during the subsequent LTM task to be much lower. Furthermore, the associative aspect of the task is likely to engage the episodic buffer, a newly added WM component which holds integrated information and may even act as an “overflow buffer” for information that may exceed the capacity of other WM stores (Baddeley, 2012).

In sum, a combined WM/LTM paradigm, which makes it possible to take either WM or LTM performance into account, appears to be particularly suited for studying which brain regions support successful execution of a WM and LTM task by reducing the “contamination” of either memory system. In other words, by implementing this kind of paradigm we were able to obtain clearer insights of the underlying neural substrates of successful WM and LTM, extending results we found in our earlier study (Bergmann et al., 2012). More general, future research needs to specify how and when performance on their WM task actually relies on WM processing rather than reflecting LTM performance or WM−LTM interactions. A combined WM−LTM task appears to be one means to account for this potential confound.

## Conflict of Interest Statement

The authors declare that the research was conducted in the absence of any commercial or financial relationships that could be construed as a potential conflict of interest.
